# Fine Mapping of a Region of Chromosome 11q23.3 Reveals Independent Locus Associated with Risk of Glioma

**DOI:** 10.1371/journal.pone.0052864

**Published:** 2012-12-31

**Authors:** Hongyan Chen, Bing Sun, Yingjie Zhao, Xiao Song, Weiwei Fan, Keke Zhou, Liangfu Zhou, Ying Mao, Daru Lu

**Affiliations:** 1 State Key Laboratory of Genetic Engineering, Fudan-VARI Genetic Epidemiology Center and MOE Key Laboratory of Contemporary Anthropology, School of Life Sciences, Fudan University, Shanghai, China; 2 Neurosurgery Department of Huashan Hospital, Fudan University, Shanghai, China; McGill University Department of Neurology and Neurosurgery, Canada

## Abstract

**Background:**

A single nucleotide polymorphism (SNP) at locus 11q23.3 (rs498872) in the near 5′-UTR of the *PHLDB1* gene was recently implicated as a risk factor for gliomas in a genome-wide association study, and this involvement was confirmed in three additional studies.

**Methodology/Principal Findings:**

To identify possible causal variants in the region, the authors genotyped 15 tagging SNPs in the 200 kb genomic region at 11q23.3 locus in a Chinese Han population-based case-control study with 983 cases and 1024 controls. We found evidence for an association between two independent loci (both the *PHLDB1* and the *ACRN1* genes) and a predisposition for gliomas. Among the multiple significant SNPs in the *PHLDB1* gene region, the rs17749 SNP was the most significant [*P* = 1.31×10^−6^ in a recessive genetic model]. Additionally, two novel SNPs (rs2236661 and rs494560) that were independent of rs17749 were significantly associated with glioma risk in a recessive genetic model [*P* = 1.31×10^−5^ and *P* = 3.32×10^−5^, respectively]. The second novel locus was within the *ARCN1* gene, and it was associated with a significantly reduced risk for glioma.

**Conclusions/Significance:**

Our data strongly support *PHLDB1* as a susceptibility gene for glioma, also shedding light on a new potentially candidate gene, *ARCN1*.

## Introduction

Gliomas that originate from glial cells are the most common primary tumors of the central nervous system (CNS), representing more than 40% of newly diagnosed brain tumors [1]. Asian populations generally show lower incidence rates than populations in Europe and North America. However, in the last few decades, China has experienced rapid increases in malignant brain tumor incidence rates, especially in large cities. In 2000, the annual incidence rate of brain tumors was less than 3.9 per 100,000 in men and 2.8 per 100,000 in women in China [2]. According to the China’s Health Statistics Yearbook from 2009, the annual mortality rate in 2004 and 2005 from gliomas was approximately 3.13 per 100,000 individuals [3]. Furthermore, glioblastoma multiforme (GBM) is the most common and the most malignant astrocytic tumor, with a median survival of only 12–15 months under the current standard of care [4]. The etiology of gliomas is largely unknown. High-dose ionizing radiation is known to increase the risk. However, only a small proportion of exposed individuals will develop gliomas, suggesting a genetic predisposition for glioma occurrence.

An association between glioma risk and the rs498872 at 11q23.3 was recently identified in a genome-wide association studies (GWAS) [5]. This association was confirmed in three additional studies [6], [7], [8], including our previous replication study in a Chinese Han population. More importantly, the SNP, which is mapped to the near 5′-UTR of *PHLDB1* (Pleckstrin homology-like domain, family B, member 1) within a 101-kb LD block on 11q23.3, is frequently deleted in patients with neuroblastoma [9]. Although there is no direct evidence of a role for this gene in gliogenesis, based on all the available data, an association between 11q23.3 and glioma appears to be one of the most consistent genetic association findings in complex diseases to date. The current data strongly suggest the presence of a glioma susceptibility locus at 11q23.3.

It is possible that other genetic variants near rs498872 in this region are responsible for the consistent risk in glioma observed for this SNP in previous studies. In this study, we performed a fine mapping study to examine the association with glioma risk of all known common sequence variants in the vicinity of the rs498872 SNP at 11q23.3.

## Results

To explore whether additional SNPs in the regions flanking SNP rs498872 are associated with glioma risk, we genotyped 15 tagging SNPs in the 200 kb genomic region at 11q23.3 in a Chinese Han study population. The distribution of selected characteristics between the 983 glioma cases and the 1,024 cancer-free controls are summarized in Table 1. Because of the frequency-matching design of this study, the distributions of age (age at diagnosis for case patients and age at inclusion for control subjects) and sex were comparable between the case patients and the control subjects. The mean age was 42.19 years for cases and 42.22 for controls. Approximately 59% of both cases and controls were male. Similar to the previous study [7], [10], [11], the cases were slightly more likely to report a family history of cancer (among first-degree relatives) than controls (17.5% versus 13.1%; *P* = 0.003). Among the cases, 306 had glioblastomas and 671 had low-grade gliomas (including 369 astrocytomas and 303 other low-grade gliomas).

**Table 1:** Characteristics of the cases and controls in a Chinese study population.

	No. of Cases	%	No. of Controls	%	P value for χ^2^ test
**Demographics**					
Total	983		1024		
**Sex**					0.545
Male	579	58.9	623	60.8	
Female	390	39.7	397	38.8	
Missing	14	1.4	4	0.4	
**Age**					0.969
Mean±SD	42.19±15.77		42.22±18.63		
**Smoke**					**0.012**
Never	587	59.7	602	58.8	
Ever	317	32.2	412	40.2	
Missing	79	8	10	0.9	
**Family History of cancer**					**0.004**
No	714	72.6	793	77.4	
Yes	160	16.3	122	11.9	
Missing	109	11.1	109	10.6	
**Histological types**					
Astrocytic glioma	369	37.6			
Glioblastoma	306	31.1			
aOther gliomas	303	30.8			
Missing	5	<1			

Bold characters indicate corresponding *P* values are less than 0.05.

a Other gliomas including oligodendrogliomas, ependymomas, ormixed gliomas.

The allele distributions of the 15 SNPs at 11q23.3 with their MAFs and associations with glioma risk are shown in Table 2. The genotype distribution in controls for all the variants were in Hardy-Weinberg equilibrium (P>0.01). To reduce the potential of spurious findings due to multiple testing, *P* = 6.67×10^−4^ (0.01/15) was considered the significance threshold using strict Bonferroni corrections [12], [13]. In the single locus analyses, we observed statistically significant differences between case patients and control participants in allele distributions for four SNPs (*P* = 2.86×10^−4^ for rs7115634, *P* = 3.32×10^−6^, for rs2236661, *P* = 2.93×10^−5^ for rs494560 and *P* = 3.08×10^−5^ for rs17748, respectively). Further logistic regression analyses revealed that patients with the rs7115634 G allele in the *ARCN1* gene had a 21% reduction of glioma risk (adjusted OR = 0.79, 95%CI = 0.70–0.89, *P* = 2.12×10^−4^). Additionally, the rs2236661, rs494560 and rs17748 in the *PHLDB1* gene were statistically significantly associated with glioma risk (adjusted *P* = 1.06×10^−5^, *P* = 4.23×10^−5^ and *P* = 2.36×10^−5^, respectively). By tumor subtypes, at a minimum separating the glioblastoma and other gliomas, three SNPs (rs2236661, rs494560 and rs17748) were significantly associated with other types (including astrocytic glioma, oligodendrogliomas, ependymomas, ormixed gliomas) but not with GBM (adjusted *P* = 9.22×10^−6^, *P* = 1.06×10^−4^ and *P* = 7.90×10^−5^, respectively).

**Table 2:** Association of 15 selected SNPs in 11q23.3 region with the risk of glioma in a Chinese population.

SNP ID	Chr.	Gene	Genomic Position	Allele	Minor Allel Frequancy			*P* value for HWE	Genotyping rate	*P* value for χ^2^	All histological types		Glioblastoma	Othere types
					Control	case	CEU				a,bOR (95% CI)	b *P* value	b *P* value	b *P* value
rs6589664	11	*TMEM25*	117910014	G:A	0.27	0.313	0.305	0.022	98.60%	5.1×10^−3^	1.22(1.05–1.43 )	6.60×10^−3^	0.078	0.01
rs12289253	11	*TMEM25*	117910278	G:A	0.48	0.508	0.844	0.089	98.60%	0.049	1.10(0.96–1.27)	0.044	0.047	0.177
rs3741324	11	*TMEM25*	117911045	G:A	0.48	0.436	0.159	0.032	98.50%	0.013	0.87(0.76–1.00)	0.011	0.034	0.058
rs10736492	11	*ARCN1*	117964479	A:G	0.24	0.258	0.429	0.23	99.10%	0.132	1.12 (0.97–1.29)	0.137	0.287	0.188
rs7115634	11	*ARCN1*	117971459	A:G	0.5	0.437	0.159	0.9	98.80%	**3.2×10** ^−**4**^	0.79 (0.70–0.89)	**2.12×10** ^−**4**^	0.012	1.39×10^−3^
rs604096	11	NA	117980308	T:C	0.29	0.267	0.261	0.081	98.30%	0.082	0.90(0.77–1.05)	0.094	0.143	0.184
rs2236661	11	*PHLDB1*	118004604	G:C	0.2	0.26	0.225	0.032	96.50%	**3.0×10** ^−**5**^	1.46 (1.23–1.72)	**1.06×10** ^−**5**^	0.019	**9.22×10** ^−**6**^
rs12419235	11	*PHLDB1*	118011767	G:T	0.16	0.172	0	0.65	97.10%	0.548	1.05 (0.88–1.24)	0.593	0.688	0.306
rs494560	11	*PHLDB1*	118026759	G:A	0.26	0.199	0.473	0.62	99.20%	**3.1×10** ^−**5**^	0.71(0.60–0.85)	**4.23×10** ^−**5**^	0.014	**1.06×10** ^−**4**^
rs17748	11	*PHLDB1*	118033634	C:T	0.27	0.325	0.237	0.2	99.40%	**3.1×10** ^−**5**^	1.36(1.17–1.59)	**2.36×10** ^−**5**^	7.41×10^−3^	**7.90×10** ^−**5**^
rs2276064	11	*TREH*	118034913	G:A	0.42	0.406	0.008	0.85	98.70%	0.582	0.94(0.81–1.08)	0.482	0.845	0.361
rs10892251	11	*TREH*	118048773	C:T	0.28	0.325	0.221	0.31	98.70%	4.6×10^−3^	1.22 (1.07–1.40)	4.12×10^−3^	0.097	7.55×10^−3^
rs11216943	11	NA	118061608	G:A	0.24	0.286	0.223	0.06	99.10%	1.52×10^−3^	1.27(1.08–1.49)	1.46×10^−3^	0.048	3.28×10^−3^
rs4639966	11	NA	118078729	T:C	0.32	0.277	0.272	0.47	98.80%	3.6×10^−3^	0.84(0.72–0.98)	4.52×10^−3^	0.018	2.59×10^−3^
rs496547	11	NA	118081673	T:A	0.25	0.253	0.358	0.49	98.00%	0.606	0.99(0.84–1.16 )	0.544	0.22	0.864

Bold characters indicate corresponding *P* values are less than 6.67×10^−4^.

a Odds ratios for the carriers of the minor allele and their associated 95% confidence intervals.

b Adjusted for age and gender.

**Figure 1:**
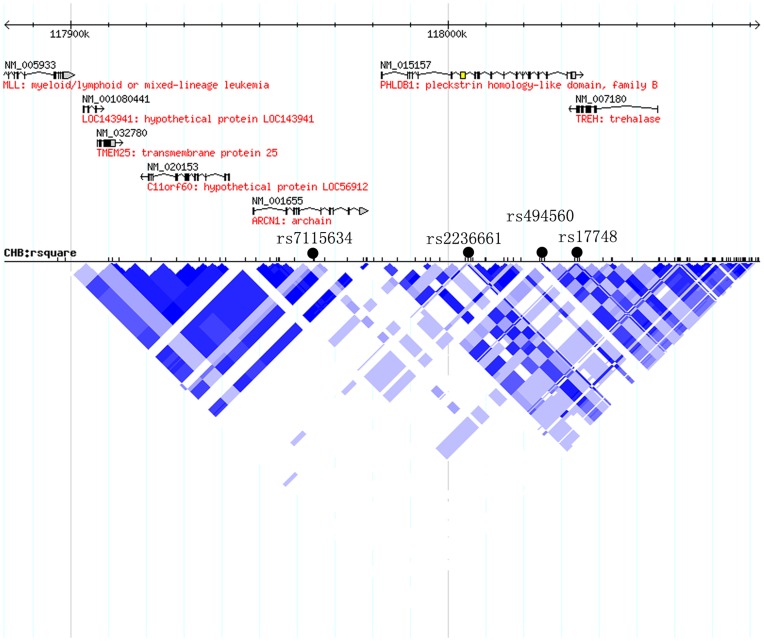
A schematic view of genetic association between SNPs at 11q23.3 and glioma risk in Chinese Han populations.

The LD plot of the chr11∶117882577–118082577 region is shown in Figure 1 (from hapmap data, release 21, Phases I and II, CHB). Two separate clusters of glioma-associated SNPs were found, including the previously reported *PHLDB1* locus and a novel locus (*ARCN1*). Among multiple significant SNPs at *PHLDB1 g*ene region, rs2236661 and rs494560 remained significantly associated with glioma risk after adjusting for rs17748 (adjusted P = 4.09×10^−6^ and P = 8.73×10^−6^, respectively), suggesting these SNPs are independent from rs17748.

Genotype frequency distributions of the four identified risk SNPs between the cases and controls are detailed in Table 3. Significant associations were observed for these four SNPs (*P* = 1.75×10^−5^ for rs2236661, *P* = 2.82×10^−4^ for rs494560 and *P* = 1.68×10^−6^ for rs17748) in a dominant model and for one SNP (*P* = 1.48×10^−4^ for rs7115634) in a recessive model, based on the best fit of the Akaike’s information criterion (Table 3). Furthermore, we performed stratified analyses by glioma histological type. The rs2236661, rs494560 and rs17748 were strongly associated with non-GBM gliomas (adjusted *P* = 2.08×10^−5^, *P* = 2.69×10^−4^ and *P* = 1.66×10^−6^, respectively).

**Table 3:** Genotype frequencies of five glioma susceptibility SNPs among cases and controls and their association with glioma risk in a Chinese population.

SNP ID	Genotype	No. of Cases	%	No. of Controls	%	All histological types		Glioblastoma		bOther types	
						aOR (95% CI)	a *P* -value	aOR (95% CI)	a *P* -value	aOR (95% CI)	a *P* -value
rs7115634	AA	290	30.1	262	25.7	1.00(reference)		1.00(reference)		1.00(reference)	
	GA	503	52.3	508	49.8	0.88(0.72–1.09)	0.236	0.95(0.70–1.28)	0.715	0.87(0.69–1.10)	0.238
	GG	169	17.6	251	24.6	0.60(0.47–0.78)	**1.16×10^−4^**	0.59(0.40–0.87)	8.31×10^−3^	0.62(0.46–0.82)	1.16×10^−3^
	GGvs.AA+GA					0.65(0.53–0.81)	**1.48×10** ^−**4**^	0.61(0.43–0.86)	5.13×10^−3^	0.68(0.53–0.87)	2.14×10^−3^
rs2236661	GG	511	54.4	632	63.5	1.00(reference)		1.00(reference)		1.00(reference)	
	GC	362	38.5	316	31.7	1.45(1.20–1.76)	**1.46×10** ^−**4**^	1.36(1.03–1.81)	0.0321	1.50(1.21–1.86)	**1.95×10** ^−**4**^
	CC	67	7.1	48	4.8	1.78(1.21–2.64)	3.81×10^−3^	1.50(0.85–2.66)	0.163	1.94(1.26–2.97)	2.41×10^−3^
	GGvs.CC+GC					1.49(1.24–1.79)	**1.75×10** ^−**5**^	1.38(1.06–1.81)	0.0191	1.56(1.27–1.91)	**2.08×10** ^−**5**^
rs494560	GG	613	63.4	565	55.2	1.00(reference)		1.00(reference)		1.00(reference)	
	GA	323	33.4	395	38.6	0.76(0.63–0.91)	3.49×10^−3^	0.84(0.64–1.10)	0.207	0.72(0.58–0.88)	2.49×**10** ^−**3**^
	AA	31	3.2	63	6.2	0.46(0.29–0.72)	**6.18×10** ^−**4**^	0.39(0.17–0.80)	0.011	0.49(0.23–0.81)	5.18×**10** ^−**3**^
	GGvs.AA+GA					0.72(0.60–0.86)	**2.82×10** ^−**4**^	0.77(0.59–1.01)	0.057	0.69(0.60–0.84)	2.69×**10** ^−**4**^
rs17748	CC	416	42.5	541	53.2	1.00(reference)		1.00(reference)		1.00(reference)	
	CT	488	49.9	413	40.6	1.54(1.28–1.85)	**4.08×10** ^−**6**^	1.30(0.98–1.71)	0.067	1.66(1.35–2.04)	4.68×**10** ^−**6**^
	TT	74	7.6	63	6.2	1.56(1.09–2.24)	0.01581	1.88(1.15–3.06)	0.012	1.56(0.95–2.17)	0.08941
	CCvs.TT+CT					1.54(1.30–1.85)	**1.68×10** ^−**6**^	1.37(1.05–1.79)	0.020	1.63(1.33–1.99)	1.66×**10** ^−**6**^

Bold characters indicate corresponding *P* values are less than 6.67×10^−4^.

a Adjusted for age and gender.

b Other types including astrocytic glioma, oligodendrogliomas, ependymomas, ormixed gliomas.

To understand the cumulative effects of these variants on glioma risks, we created a variable to combine the effects of risk alleles per individual from the four independent risk variants (rs7115634, rs2236661, rs494560 and rs71148). Overall, glioma risk increased with increasing numbers of risk variant alleles. Individuals carrying 6–8 risk alleles had a 1.93-fold increased risk of developing glioma compared with those who carried 0–2 risk alleles (adjusted OR = 1.93, 95% CI = 1.46 to 2.55, P = 3.70×10^−6^, Table 4).

**Table 4:** Association between the cumulative effect of four significant SNPs^a^ and the risk of glioma in a Chinese population.

Number of risk alleles	No. of Cases (%)	No. of Controls (%)	OR(95% CI)	b *P* value
0–2	231 (23.5)	348 (34.0)	1.00(reference)	
3	191 (19.4)	206 (20.1)	1.35(1.04–1.75)	**0.023**
4	224 (22.8)	192 (18.8)	1.77(1.37–2.28)	**1.28×10** ^−**5**^
5	160 (16.3)	138 (13.5)	1.76(1.32–2.34)	**9.77×10** ^−**5**^
6–8	177 (18.0)	140 (13.7)	1.93(1.46–2.55)	**3.70×10** ^−**6**^

Bold characters indicate corresponding *P* values are less than 6.67×10^−4^.

a Significant SNPs including s7115634, rs2236661, rs494560 and rs17748.

b Adjusted for age and gender.

**Figure 2:**
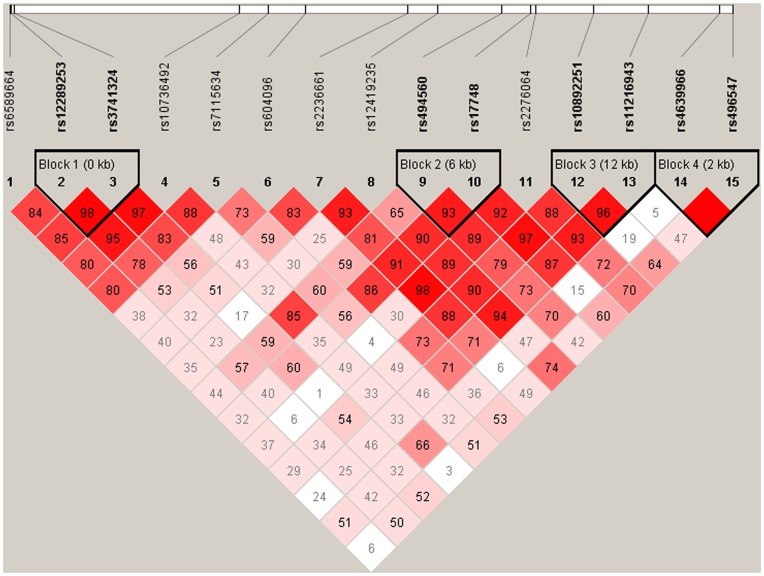
LD plot of 11q23.3 using 15SNPs in 1024 ethnic Han Chinese controls. This plot was generated by the Haploview program with four Gamete Rule setting. Four blocks were determined. The rs number (top; from left to right) corresponds to the SNP name and the level of pairwise D’ indicates the degree of LD between the two SNPs.

Figure 2 shows plots of the pairwise LD (D’) values for the 15 SNPs and the LD structure of the 11q23.3 region. Four blocks were defined by 8 SNPs (rs12289253 and rs3741324; rs494560 and rs17748; rs10892251 and rs11216943; and rs4639966 and rs496547). Global score tests showed statistically significant differences in haplotype frequency distributions between the cases and the controls for block 2 and block 3 (*P*_sim_ = 5.0×10^−6^) but not for block 1 and block 4 (Table 5).

**Table 5:** Frequency distributions of haplotypes in 11q23.3 region among cases and controls, and their associations with glioma risk in a Chinese population.

a11q23.3 Blocks	Haplotype	Case	Control	*P*	b,c *P*-sim	bOR(95% CI)
Block1	AG	0.502	0.476	0.078	0.083	1.000(reference)
	GA	0.434	0.472	0.011	0.013	0.856(0.747–0.981)
	GG	0.06	0.049	0.141	0.137	1.154(0.868–1.535)
	AA	0.004	0.003			
	Global score = 7.64 *df = *3 *P* = 0.0541 c *P_-sim_* = 0.0538					
Block2	GC	0.476	0.484	0.679	0.68	1.000(reference)
	GT	0.324	0.261	**8.49×10^−6^**	**5.00×10^−6^**	1.297(1.114–1.511)
	AC	0.199	0.251	**3.75×10^−5^**	**6.50×10^−5^**	0.796(0.676–0.937)
	AT	0.001	0.004			
	Global score = 28.77 *df = *3 *P* = **2.5×10^−5^** c *P_-sim_* = **5.0×10^−6^**					
Block3	CG	0.655	0.71	**1.0×10^−4^**	**9.00×10^−5^**	1.000(reference)
	TA	0.267	0.237	0.019	0.019	1.270(1.092–1.479)
	TG	0.058	0.046	0.098	0.098	1.400(1.047–1.873)
	CA	0.02	0.007			
	Global score^ = ^25.53 *df = *3 *P* = **1.2×10^−5^** c *P_-sim_* = **5.0×10^−6^**					
Block4	TT	0.469	0.434	0.027	0.028	1.000(reference)
	CT	0.276	0.32	0.003	0.004	0.795(0.684–0.923)
	TA	0.253	0.246	0.566	0.57	0.952(0.815–1.113)
	CA	0.001				
	Global score = 10.59 *df* = 3 *P* = 0.0141 c *P_-sim_*_ = _0.0087					

Bold characters indicate corresponding *P* values are less than 6.67×10^−4^.

a Order of polymorphisms is rs12289253, rs3741324 in the block1; rs494560, rs17748 in the block2; rs10892251,rs11216943 in the blok3; rs4639966,rs496547 in the block4.

b Adjusted for age and gender.

c Generated by the permutation test with 10,000 times simulations.

## Discussion

To explore the contribution of genetic variation at the 11q23.3 locus that was previously identified by GWAS and our previous replication study for gliomas, we performed mapping of this region, including the *PHLDB1* gene, using a highly correlated tag SNP approach. After genotyping 15 tagged SNPs from a 200 -kb region of LD flanking the initial SNP marker (rs498872), rs17748 was in strong LD with rs498872 (r^2^ = 0.826) and was significantly associated with glioma risk. Additionally, we found two novel independent SNPs (rs7115634 and 2236661) in the *PHLDB1* gene and one SNP (rs494560) in the *ARCN1* gene that conferred to glioma risk.

PHLDB1 (also known as LL5α) is a protein that was first identified in a bioinformatics screen [14]. It contains a Forkhead-associated (FHA) domain and a C-terminal PH domain [15]–[17]. The database from the Genomics Institute of the Novartis Research Foundation shows that PHLDB1 is highly expressed in the brain and adipose tissues of mice and humans. Although the PH domain of PHLDB1 possesses a potential PI(3,4,5)P_3_-binding motif, the molecular basis by which PH domains are able to interact with PI(3,4,5)P_3_ has not been established definitively. Recently, Zhou *et al.* [18] demonstrated that PHLDB1 binds PI(3,4,5)P_3_ through its PH domain and that PHLDB1 functions in adipocytes as a positive regulator of Akt activation, where it is required for optimal insulin induced glucose transport and GLUT4 translocation. However, there is no direct functional evidence of a role for PHLDB1 in the initiation of tumors.

Through fine-mapping, the strongest signal was still located on the *PHLDB1* gene. In our study, the rs223661 C allele and the rs17748 T allele exhibited a statistically significant increased risk of glioma (OR = 1.46, 95% CI = 1.23 to 1.72, *P* = 1.06×10^−5^ and OR = 1.36, 95% CI = 1.17 to 1.59, *P* = 2.36×10^−5^, respectively), and the rs494560 A allele showed a significantly protective effects (OR = 0.71, 95% CI = 0.60 to 0.85, *P* = 4.23×10^−5^). Individuals with the ‘GT’ haplotype, which consists of the containing the ‘G’ risk allele of rs494560 and the ‘T’ risk allele of rs17748, had a 1.30-fold higher risk of developing glioma than individuals with the ‘GC’ haplotype, which was consistent with the individual SNP association analysis. Rs17748, which is located in the 3′UTR of *PHLDB1,* and the previously reported rs498872 were in strong LD (r^2^ = 0.826). Thus, the significant association between these SNPs and gliomas risk appeared to be consistent between our two separate studies. It is notable that rs17748 was also reported to be significantly associated with glioma risk in a European populations by a recent GWA study [19], [6]. Those data strongly suggest that rs17748 and rs498872 are involved with the etiology of gliomas. Importantly, rs2236661 and rs494560 remained significantly associated with glioma risk after adjusting for rs17748 (adjusted *P* = 4.09×10^−6^ and *P* = 8.73×10^−6^, respectively), suggesting that these SNPs have a role that is independent from rs17748.

Recent advances in understanding of glioma subtypes (e.g. proneural, neural, mesenchymal) based on gene expression [20], somatic mutations (e.g. IDH1) [21] and global patterns of methylation (glioma CpG island methylator pheynotype; G-CIMP) [22] suggest there are important subgroups of glioma that may represent distinct pathological entities. Jenkins et al. [8] report that specific germ line polymorphisms are associated with different glioma subtypes. Similar to previously reports, we note that 3 SNPs (rs 226661, rs494560 and rs17748) in *PHLDB1* gene was only strongly associated with other non-GBM gliomas but not with GBM.

Additionally, we also detected a novel association that is independent of *PHLDB1*. Rs7115634 was in low LD (pairwise r^2^<0.122, Table S1) with rs17748, rs2236661, and rs494560 in our Chinese population. This newly identified maker SNP is mapped to the intron of *ARCN1*, which is located within the commonly deleted region of neuroblastoma patients at human chromosome 11q23.3 [14].

ARCN1, also known as δ-COP, is a sub-unit of the coat protein I (COPI) complex binds to dilysine motifs and reversibly associates with Golgi non-clathrin-coated vesicles. The association further mediates biosynthetic protein transport from the ER via the Golgi up to the trans-Golgi network [23]–[26]. The mutation in *ARCN1* results in phenotypes commonly observed in neurodegenerative disorders, such as abnormal protein accumulation, ER stress, and neurofibrillary tangles [27]. TCGA (The Cancer Genome Atlas) database (http://cancergenome.nih.gov/cancersselected/glioblastomamultiforme) showed that ARCN1 expression was increased in glioblastomas. However, the exact mechanism of the *ARCN1* gene in the development of glioma still needs further investigation.

Nevertheless, it is worth noting that the three newly identified variants (rs7115634 in *ARCN1*, rs2236661 and rs494560 in *PHLDB1*) and the rs17748 SNP contributed to a cumulative risk effect for glioma susceptibility. Individuals carrying 6–8 risk alleles had a 1.93-fold increased risk of developing gliomas when compared with those who carried between 0 and 2 risk alleles, indicating the importance of combined effects from independent risk variants in the etiology of glioma.

The genetic variants within conventional regulatory regions, such as the 5′UTR and the 3′UTR, were given priority in most previous studies; however, accumulating evidence indicates the importance of intronic polymorphisms as markers of disease susceptibility [28]–[30]. It is possible that all identified SNPs (rs17748 in the 3′UTR, rs7115634, rs2236661 and rs494560 in introns) in this region affect glioma risk by modulating *PHLDB1* and *ARCN1* expression levels or function. However, these hypotheses are based on speculation, and they need to be confirmed by biological assays in future studies.

In summary, through fine-mapping of the 11q23.3 region in a large sample of Chinese population, we identified a novel marker in the *ARCN1* gene and two new variants in the *PHLDB1* gene. These markers are individually related to the susceptibility to glioma in this population. Further functional evaluation and larger association studies with ethnically diverse populations are needed to elucidate the role of these causative or marker SNPs in the development of glioma.

## Materials and Methods

### Study Population

Using the same recruitment method described elsewhere [8], [10], [11], [31], we recruited 983 patients with histopathologically confirmed gliomas and 1,024 healthy controls between October 2004 and July 2009 from the Department of Neurosurgery at Huashan Hospital, Fudan University (Shanghai, China). There were no restrictions on age, sex, or histologic type, but patients with a self-reported history of cancer and patients with previous radiotherapy or chemotherapy for unknown disease conditions were excluded. Additionally, diagnoses of potentially eligible cases were validated by trained abstractors who reviewed the pathologic and medical records of all cases to confirm that there were no undiagnosed occult primary tumors at the time of recruitment. All controls were frequency-matched to the cases by age (within 5 years), sex, and residential area (urban area or countryside). The controls were recruited from visitors to the trauma outpatient clinic and from persons undergoing annual check-ups at the same hospital. These controls had no known central nervous system-related diseases, no self-reported history of cancer at any site, and no history of radiotherapy/chemotherapy for unknown disease conditions. No evidence of demographic differences was found between the trauma outpatients and the annual check-up subjects. All cases and controls were from Shanghai and the surrounding provinces (Zhejiang, Jiangsu, and Anhui) in eastern China, and all were of Han Chinese ethnic background.

Written informed consent was obtained from all participants or from the patients’ representatives. Each subject was interviewed face-to-face by trained personnel using a previously described questionnaire [11] to obtain demographic data, history of environmental exposure to ionizing radiation and overall health characteristics. After the interview, each subject provided 3–5 mL of venous blood. From all of the participants, blood samples and questionnaires were available for 983 cases and 1024 control subjects, representing a 92.6% and 88.2% of all eligible case and control subjects, respectively.

### Selection of Tagging SNPs and Genotyping

CHB data were obtained from the International HapMap Project (http://www.hapmap.org) using the phase II Nov 08, on NCBI B36 assembly, dbSNP b126 and the phase III Aug 10, on NCBI B36 assembly, dbSNP b132. The minor-allele frequency (MAF) was ≥0.05, the Hardy–Weinberg equilibrium (HWE) cutoff was ≥0.05 and the call rate was ≥95%. Based on a block-based tagging strategy using HaploView program 4.2, we targeted a 200 kb region (chr11∶117882577–118082577, 200,000) that included the *MLL* (myeloid/lymphoid or mixed–lineage leukemia), *TMEM25* (transmembrane protein 25), *ARCN1* (archain), *PHLDB1*, and *TREH* (trehalase) genes. A total of 17 tagging SNPs were identified to capture (r^2^≥0.8) all SNPs (119 SNPs) with minor allele frequencies of at least 5% in this region. Two SNPs (rs576950 and rs633308) could not be assayed using our technique. The previously reported rs498872 SNP was not included in this tagging SNP panel because of the overlap in patient populations between the two studies. However, rs498872 was well tagged by rs17748 in this study (r^2^ = 0.826).

Genomic DNA was extracted from leukocytes using the Qiagen Blood Kit (Qiagen, Chatsworth, California, USA). Then, the genomic DNA was diluted to a final concentration of 15–20 ng/µl for the subsequent assays. Primers for amplification and extension reactions were designed using the MassARRAY Assay Design software, version 3.1 (Sequenom, San Diego, California). Polymorphism-flanking fragments were amplified by polymerase chain reaction. Genotyping was performed on the Mass-ARRAY iPLEX platform (Sequenom) through the use of an allele-specific matrix-assisted laser desorption/ionization time-of-flight mass spectrometry assay [32] without knowing the case or control status. All assays were carried out in 384-well arrays, and 8 blank controls and 8 random duplicates were used for quality control. The results were more than 98% concordant between the duplicated samples. On average, 98% of the genotypes were successfully assayed for all SNPs.

### Statistical Analyses

We used Fisher’s exact test to test for deviations from Hardy-Weinberg equilibrium among the controls for each SNP. We used the χ^2^ test to examine the differences in demographic characteristics and frequency distributions of genotypes and alleles between the cases and controls. The most common genotype in the controls was used as the reference group. We performed unconditional logistic regression analysis with adjustments for age, sex, cigarette smoking and family history of cancer to calculate odds ratios and 95% confidence intervals of the estimates of the relative risk for each SNP and for multiple SNPs. All statistical tests were two-sided. Akaike’s information criteria (AIC) were employed to determine the best fitting model for each SNP [33].

We also evaluated the cumulative effects of the risk alleles, which were independently associated with glioma risk, by counting the total number of risk alleles per individual from the four independent risk variants (rs7115634, rs2236661, rs494560 and rs71148) (categories 0–2, 3, 4, 5, and 6–8). Statistical analyses were all performed using the SPSS17.0 software, unless indicated otherwise.

Pairwise LD parameter D’ was calculated using the Haploview program (http://www.broad.mit.edu/mpg), and D’ >0.8 was defined for 2 SNPs in strong LD [34]. The Haplo.Stats package which runs in R environment was used to infer the haplotypes of the genotyped SNPs using the “haplo.glm” function, and an additive model was assumed to estimate the haplotype specific OR with adjustments for possible confounding variables (i.e., age, sex). The “haplo.score” function was introduced to calculate the global and haplotype-specific permutation *P* value (P_sim_, minimal simulation: 10,000 with a significance level less than 0.05) [35].

### Ethics

Written informed consent was obtained from each participant, and the study was approved by the School of Life Sciences of Fudan University Ethics Board.

## Supporting Information

Table S1 **Pairwise linkage disequilibrium (D’, r^2^) between of 15 SNPs in 11q23.3 region.**
(DOC)

Table S2 **Interaction between pairs of SNPs in 11q23.3 region.**
(DOC)
