# Supplementary material for: Fine Mapping of a Region of Chromosome 11q23.3 Reveals Independent Locus Associated with Risk of Glioma
Source: PLoS One. 2012 Dec 31;7(12):e52864. doi: 10.1371/journal.pone.0052864 (PMC3534108; doi:10.1371/journal.pone.0052864)
Supplement: Table S1 — Pairwise linkage disequilibrium (D’, r2) between of 15 SNPs in 11q23.3 region. (DOC) [file pone.0052864.s001.doc]

**Table S1. Pairwise linkage disequilibrium (D’, r^2^) between of 15 SNPs in 11q23.3 region**

|  | rs7445640 | rs13361701 | rs4073918 | rs2736118 | rs4246742 | rs4975605 | rs10069690 | rs2735948 | rs2853668 | rs4975612 | rs4635969 | rs6554759 | rs401681 | rs414965 |
| --- | --- | --- | --- | --- | --- | --- | --- | --- | --- | --- | --- | --- | --- | --- |
| rs7445640 | － | 1.000 | 0.873 | 0.596 | 0.362 | 0.147 | 0.211 | 0.029 | 0.326 | 0.153 | 0.410 | 0.076 | 0.146 | 0.266 |
| rs13361701 | 0.001 | － | 0.736 | 0.299 | 0.309 | 0.598 | 1.000 | 0.294 | 1.000 | 0.344 | 0.223 | 0.036 | 0.369 | 0.183 |
| rs4073918 | 0.609 | 0.005 | － | 0.599 | 0.395 | 0.145 | 0.326 | 0.013 | 0.384 | 0.136 | 0.429 | 0.096 | 0.152 | 0.221 |
| rs2736118 | 0.063 | 0.006 | 0.051 | － | 0.529 | 0.081 | 0.130 | 0.068 | 0.020 | 0.256 | 0.160 | 0.021 | 0.267 | 0.002 |
| rs4246742 | 0.026 | 0.000 | 0.039 | 0.010 | － | 0.253 | 0.456 | 0.150 | 0.042 | 0.057 | 0.413 | 0.097 | 0.087 | 0.300 |
| rs4975605 | 0.006 | 0.000 | 0.005 | 0.004 | 0.010 | － | 0.416 | 0.229 | 0.587 | 0.327 | 0.282 | 0.598 | 0.287 | 0.281 |
| rs10069690 | 0.026 | 0.001 | 0.050 | 0.005 | 0.024 | 0.003 | － | 0.117 | 0.113 | 0.202 | 0.605 | 0.296 | 0.264 | 0.013 |
| rs2735948 | 0.000 | 0.002 | 0.000 | 0.002 | 0.006 | 0.029 | 0.012 | － | 0.785 | 0.057 | 0.593 | 0.704 | 0.591 | 0.624 |
| rs2853668 | 0.014 | 0.002 | 0.025 | 0.000 | 0.001 | 0.013 | 0.001 | 0.043 | － | 0.885 | 0.785 | 0.524 | 0.653 | 0.699 |
| rs4975612 | 0.008 | 0.000 | 0.008 | 0.004 | 0.002 | 0.010 | 0.008 | 0.001 | 0.320 | － | 0.292 | 0.634 | 0.435 | 0.027 |
| rs4635969 | 0.006 | 0.002 | 0.008 | 0.000 | 0.030 | 0.070 | 0.008 | 0.223 | 0.027 | 0.009 | － | 0.532 | 0.945 | 0.975 |
| rs6554759 | 0.001 | 0.000 | 0.001 | 0.000 | 0.000 | 0.002 | 0.022 | 0.141 | 0.005 | 0.019 | 0.001 | － | 0.979 | 1.000 |
| rs401681 | 0.016 | 0.001 | 0.022 | 0.002 | 0.006 | 0.018 | 0.006 | 0.136 | 0.077 | 0.085 | 0.222 | 0.107 | － | 0.983 |
| rs414965 | 0.004 | 0.001 | 0.003 | 0.000 | 0.026 | 0.044 | 0.000 | 0.383 | 0.035 | 0.000 | 0.598 | 0.280 | 0.382 | － |

D’, Standardized linkage disequilibrium coefficient (above diagonal line).

r^2^, Squared correlation coefficient (below diagonal line).
