# Supplementary material for: Fine Mapping of a Region of Chromosome 11q23.3 Reveals Independent Locus Associated with Risk of Glioma
Source: PLoS One. 2012 Dec 31;7(12):e52864. doi: 10.1371/journal.pone.0052864 (PMC3534108; doi:10.1371/journal.pone.0052864)
Supplement: Table S2 — Interaction between pairs of SNPs in 11q23.3 region. (DOC) [file pone.0052864.s002.doc]

**Table S2. Interaction between pairs of SNPs in 11q23.3 region**

| Variation | rs7115634 | rs2236661 | rs494560 | rs17748 |
| --- | --- | --- | --- | --- |
| rs7115634 | - |  |  |  |
| rs2236661 | 0.74 | - |  |  |
| rs494560 | 3.42×10^-6^ | 0.86 | - |  |
| rs17748 | 0.75 | 4.21×10^-5^ | 0.673 | - |
